# EBI2 receptor regulates myelin development and inhibits LPC-induced demyelination

**DOI:** 10.1186/s12974-017-1025-0

**Published:** 2017-12-16

**Authors:** Aleksandra Rutkowska, Andreas W. Sailer, Kumlesh K. Dev

**Affiliations:** 1 Drug Development, School of Medicine, Trinity College, Dublin, Ireland; 2 Chemical Biology & Therapeutics, Novartis Institutes for BioMedical Research, Novartis Pharma AG, Basel, Switzerland; 3 Medical University of Gdańsk, M. Skłodowskiej-Curie 3a, Gdańsk, Poland

**Keywords:** Epstein-Barr virus-induced gene 2 (EBI2 receptor), Multiple sclerosis, Myelination, Demyelination, Organotypic slices, Neuroinflammation

## Abstract

**Background:**

The G protein-coupled receptor EBI2 (Epstein-Barr virus-induced gene 2) is activated by 7α, 25-dihydroxycholesterol (7α25HC) and plays a role in T cell-dependant antibody response and B cell migration. Abnormal EBI2 signaling is implicated in a range of autoimmune disorders; however, its role in the CNS remains poorly understood.

**Methods:**

Here we characterize the role of EBI2 in myelination under normal and pathophysiological conditions using organotypic cerebellar slice cultures and EBI2 knock-out (KO) animals.

**Results:**

We find that MBP expression in brains taken from EBI2 KO mice is delayed compared to those taken from wild type (WT) mice. In agreement with these in vivo findings, we show that antagonism of EBI2 reduces MBP expression in vitro. Importantly, we demonstrate that EBI2 activation attenuates lysolecithin (LPC)-induced demyelination in mouse organotypic slice cultures. Moreover, EBI2 activation also inhibits LPC-mediated release of pro-inflammatory cytokines such as IL6 and IL1β in cerebellar slices.

**Conclusions:**

These results, for the first time, display a role for EBI2 in myelin development and protection from demyelination under pathophysiological conditions and suggest that modulation of this receptor may be beneficial in neuroinflammatory and demyelinating disorders such as multiple sclerosis.

**Electronic supplementary material:**

The online version of this article (10.1186/s12974-017-1025-0) contains supplementary material, which is available to authorized users.

## Background

EBI2 (Epstein-Barr virus induced gene 2, aka GPR183) is a G protein coupled receptor involved in regulation of T cell-dependent antibody response in B cells [1, 2]. The receptor’s most potent endogenous agonist is an oxysterol, 7α, 25-dihydroxycholesterol (7α25HC). Oxysterols, oxygenated derivatives of cholesterol, are involved in cholesterol homeostasis and turnover and have been shown to play important roles in immune defense, astrocyte biology, as well as in myelination [3–7]. Cholesterol is a crucial component of myelin, and deficiency or lack of cholesterol staggers the rate of myelin formation and results in motor symptoms, as has been shown in genetically modified mice in which myelinating oligodendrocytes cannot synthesize cholesterol [8]. In addition to being involved in normal myelin development, cholesterol and its metabolites, in particular oxysterols, have also been shown to have detrimental effects on myelin. For instance, they inhibit myelin gene expression in the peripheral nervous system via liver x receptor (LXR) α and β mediated signaling [9]. Oxysterols have also been shown to have adverse effects on oligodendrocyte viability in vitro*.* Indeed, treatment of oligodendrocyte cell line 158N with 25HC or 22SHC induced cell death and morphological changes independently of LXR signaling [10]. On the other hand, mass spectroscopy analysis of oxysterols showed the presence of oxysterol biosynthetic enzymes and oxysterols in oligodendrocytes indicating that oxysterols may signal in an autocrine/paracrine manner in oligodendrocytes [10]. Moreover, oxysterols enhance expression and activity of phospholipase A2 leading to a protective effect in oligodendrocytes [10]. These studies show that oxysterols may play dual role in oligodendrocyte/CNS biology having either detrimental or supportive functions depending on the oxysterol [10].

Abnormal levels of EBI2 and oxysterols have been found in a number of neurodegenerative diseases such as multiple sclerosis (MS), Alzheimer’s disease, or cerebrotendinous xanthomatosis [11–14]. For instance, EBI2 was shown to be functionally expressed in MS patients [15]. Specifically, EBI2 is highly expressed in infiltrating Th17 cells in MS lesions [16]. Moreover, its expression is particularly enhanced in memory CD4+ T cells in Natalizumab-treated MS patients [15]. On the other hand, altered levels of oxysterols have been detected in experimental autoimmune encephalomyelitis (EAE) animals, the animal model of MS, as well as in MS patients [14, 17]. For instance, plasma levels of 25HC, the precursor of 7α25HC, were reduced in relapsing-remitting MS patients compared to controls [14]. It has been proposed that lower synthesis of this oxysterol by macrophages decreased the negative-feedback loop exerted by 25HC on IL1-family of cytokines leading to aggravated MS course [14]. This hypothesis is based on a study where deficiency of CH25H in mice resulted in exaggerated course of EAE and also lead to overproduction of IL1β in CH25H KO macrophages suggesting anti-inflammatory properties of 25HC [18]. However, opposite findings were also reported in CH25H KO mice where attenuated course of EAE was observed [19]. In this study, lower levels of CH25H resulted in decreased 7α25HC synthesis leading to diminished trafficking of pathogenic CD4+ T cells to the CNS [19]. Similar findings were reported in cells derived from childhood cerebral X-linked adrenoleukodystrophy patients [20]. In these patients, increased production of 25HC by pluripotent stem cells and fibroblasts was found to induce NLRP3 inflammasome signaling. Moreover, in mice injected with 25HC in the corpus calossum recruitment of microglia, increased levels of IL1β and oligodendrocyte death was reported [20]. Another study, however, found that during EAE, genes such as CH25H are upregulated in microglia during demyelinating and remyelinating stages of the disease to support myelination [21]. Similarly, a study has shown that treatment of mouse astrocytes with LPS induces the release of 25HC, 7α25HC, and 7β25HC from these cells [6]. Furthermore, this LPS-conditioned media induced macrophage migration indicating an important role EBI2/oxysterols axis plays in the crosstalk between the immune and CNS cells. Of interest, mutations in the CYP7B1 gene, which encodes for the enzyme that converts 25HC into a 7α25HC, have been identified as the cause of spastic paraplegia gene 5 (SPG5). This neurodegenerative disorder belongs to a group of diseases called hereditary spastic paraplegias and is characterized by neuropathy of upper motor neurons as well as periventricular and subcortical white matter lesions [22, 23].

Inflammation in the CNS and the presence of pro-inflammatory cytokines such as TNFα/β and IL1β have been shown to induce demyelination in vivo [24, 25]. Direct injection of TNFα/β into the sciatic nerve induced inflammation, axonal damage, and demyelination [24]. In another study, chronic IL1β expression induced inflammation, breakdown of the BBB, activation of astrocytes and microglia, and pronounced demyelination without the loss of neurons [25]. EBI2 expression in Th17 cells was shown to be regulated by IL1β and IL23 cytokines [16]. Importantly, enhanced EBI2 expression in these cells promoted passive EAE. Inflammation and presence of pro-inflammatory cytokines and oxidative stress have been shown to induce demyelination also in organotypic slice cultures [26, 27]. Studies have shown that lysophosphatidylcholine (LPC) and lipopolysaccharide (LPS) induce increase in IL1β, IL6, TNFα, CXCL5 (LIX), and CCL3 (MIP1α) in organotypic slices and lead to oligodendrocyte and axonal damage and demyelination [26, 27]. Previous studies have suggested that inhibition of pro-inflammatory chemokine/cytokine levels in cerebellar slices might be a mechanism via which demyelination is restricted [27–29]. Treatment of cerebellar slices with FTY720 or BAF312, S1P receptors agonists with immunomodulatory properties, attenuated LPC-or galactosylsphingosine (psychosine)-induced demyelination and pro-inflammatory chemokine release [27–29]. Similar mechanisms were observed for cuprizone-induced demyelination in corpus callosum where treatment with FTY720 protected from demyelination via inhibition of pro-inflammatory cytokine release such as IL1β [30]. Others have also shown that inhibition of TNFα signaling attenuated demyelination in organotypic slices indicating a direct role of pro-inflammatory cytokines in mediating myelin damage [26].

Since oligodendrocytes use cholesterol as a building block for myelin, and oxysterols are involved in cholesterol homeostasis and turnover, it is possible that EBI2, which is activated by oxysterols, may play an important role in myelination [3]. Thus, to examine the role of EBI2 signaling in myelination under normal and pathophysiological conditions, the effect of EBI2 activation on myelination state was examined in vitro using organotypic cerebellar slices and in vivo in wild type (WT) and EBI2 KO animals.

## Methods

### Compounds, antibodies, and stains

EBI2 antagonist (NIBR189) [31] and 7α25HC were prepared as 10 mM stock solutions dissolved in 90% dimethyl sulfoxide (DMSO, Sigma). Lysolecithin from egg yolk (L-α-Lysophosphatidylcholine, LPC, Sigma, L4129) was prepared as 25 mg/ml stock in serum-free media. Primary rabbit polyclonal antibodies were anti-glial fibrillary acidic protein (GFAP, Abcam, ab7260), anti-myelin basic protein (MBP, Abcam, ab40390), anti-Iba1 (Wako, 019-19741), and anti-actin (Pierce, PA1-16889). Primary monoclonal anti-human EBI2 antibodies were generated by genetic immunization of mice [32] and made available through Euroscreeen S.A. Belgium. One hybridoma subclone 607B1C31C1 (batch ACE25368), known as 607B, was used. Primary rat monoclonal antibodies were anti-CD31 (BD, 553370) and anti-F4/80 (eBiosciences, 14-4801-81). Mouse monoclonal antibodies used were anti-MBP (Covance, SMI 94), anti-olig1 (Millipore, MAB5540), anti-CNPase (Millipore, MAB326R), and anti-MOG (Millipore, MAB5680). The other primary antibody used in this study was chicken polyclonal anti-neurofilament heavy chain (Millipore, AB5539). Secondary goat antibodies used for immunocytochemistry were anti-mouse 488 (Invitrogen Alexa, A11029), anti-mouse 633 (Invitrogen Alexa, A21052), anti-rabbit 488 (Invitrogen Alexa, A11008), anti-rabbit 633 (Invitrogen Alexa, A21070), anti-chicken 633 (Invitrogen Alexa, A21103), anti-rat 488 (Invitrogen Alexa, A-11006), and anti-rat 633 (Invitrogen Alexa, A21094). Human secondary mouse antibody used was anti-human 488 (Invitrogen Alexa, A10631). Other secondary antibodies used were Jackson ImmunoResearch donkey anti-chicken 549 (703-505-155) and anti-mouse 549 (715-505-020). Secondary antibodies used for Western blotting (WB) and conjugated to horse-radish peroxide (HRP) were donkey anti-rabbit IgG (GE Healthcare, NA934) and goat anti-mouse (Sigma, A8924). Dye used for cellular staining was nuclear stain Hoechst 34580 (Invitrogen, H21486).

### Animal husbandry

The EBI2 double (−/−) KO mouse strain (mixed genetic background C57BL/6 x C129) was purchased from Deltagen [33]. All mice, the KO and WT littermates, were housed in filter-top cages under specified pathogen-free conditions, and a standard diet and water were provided ad libitum. All animal procedures were approved by the institutional ethics committee (Novartis Pharma, Basel, Switzerland, and Trinity College Dublin, Ireland). For myelin development experiments, WT and EBI2 KO animals were sacrificed at indicated time points; whole brains were removed from skulls and snap frozen in liquid nitrogen. Heads of embryonic day 14 (E14) animals were detached and frozen in liquid nitrogen with the skin and remaining tissue.

### Tissue and cell culture

Cerebellar slice cultures were prepared, using brain tissue isolated from postnatal day 10 WT C57BL/6 mice or EBI2 KO mice according to the protocol reported earlier [27–29, 34, 35]. Briefly, mice were decapitated, cerebellar tissue was removed from the skull, cooled down in Opti-MEM (Invitrogen, 11058021), and separated from hindbrain with spatulas. Cerebellum was cut into 400 μm parasagittal slices using a McIlwain tissue chopper. Slices were cooled in Opti-MEM on ice and later separated into individual slices under dissection microscope using needles. Four to six slices were grown on each cell culture insert (Millicell, PICMORG50). Slices were cultured using an interface method with 1 ml of medium per 35 mm well. Slices were grown in 50% Opti-MEM, 25% Hanks’ buffered salt solution (HBSS, Invitrogen, 14025-050), 25% heat-inactivated horse serum (Bio-Sera, HO-290) supplemented with 2 mM Glutamax (Invitrogen, 35050-038), 28 mM D-glucose (Sigma, G8769), 1% pen/strep, and 10 mM HEPES (Sigma, H3784) for 12–14 days in vitro unless otherwise indicated in figure legends. Slice cultures were grown at 35.5 °C and 5% CO_2_ in a humidified incubator. Prior to treatment, slices were starved in serum free media for 4 h and then transferred to fresh serum-free medium containing LPC (0.4 mg/ml) (Sigma, L-4129) and incubated overnight for 18 h, with or without 7α25HC (1 μM) and/or NIBR189 (NIBR189, 1 μM). Following 18 h LPC, 7α25HC and/or NIBR189 treatment, slices were transferred to medium containing 7α25HC (1 μM) and NIBR189 (1 μM) alone for further 30 h (48 h in total). For myelin development experiments, slices were grown in 7α25HC (1 μM) or NIBR189 (1 μM) or control media immediately after preparation for 7, 14, or 20 days in vitro (DIV). Fresh compounds were added every other day when media was changed. For myelin development in WT versus EBI2 KO animals, slices were grown in complete media without compounds for indicated time. To measure the extent of neuronal demyelination, immunocytochemistry was performed. Human monocyte (U937) was grown in RPMI 1640 medium supplemented with Glutamax, 10% FBS, 1% sodium pyruvate (Invitrogen, 11360-070), 1% MEM non-essential amino acids (NEA, Invitrogen, 11140), 0.1% 2-Mercaptoethanol (Sigma, M7154), and 1% pen/strep in T75 culture flasks. Cells were grown in a humidified incubator at 37 °C and 5% CO_2_.

### Enzyme-linked immunosorbent assays

Mouse cerebellar slices were treated as described above, and media was collected following 18 h LPC treatment with or without the compounds treatment and frozen at − 20 °C. Levels of cytokines in the supernatant were measured with the following RnD Systems ELISA kits: mouse IL6 (DY406) and mouse IL1β (DY401) according to the manufacturer’s instructions and similar to our previous reports [28, 29, 36]. Briefly, 96-well ELISA plates (Thermo Scientific, 95029780) were coated overnight at room temperature with capture antibodies diluted in PBS. The plates were washed three times with wash buffer (0.05% Tween 20 (Sigma, P7949), PBS, pH 7.4) and then blocked for 1 h at room temperature with 1% BSA. The plates were then washed three times with wash buffer, and any remaining buffer was removed from the wells by aspiration. A standard curve was prepared using serial dilutions of the recombinant protein diluted in their reagent diluents: 1% BSA in PBS or for mouse IL1β 0.1% BSA, 0.05% Tween-20 in tris-buffered saline (TBS, Fisher, 10785341). The samples and standards were incubated in the antibody-coated ELISA plate for 2 h at room temperature. The plate was then washed three times with wash buffer, and 50 μl of the detection antibody (diluted in their respective reagent diluents) was added to all wells. Following three further washes, 50 μl of streptavidin-HRP diluted in reagent diluents was added to each well and incubated for 20 min at room temperature, protected from light. Three further washes were carried out, and the wells were incubated with 50 μl of substrate solution (RnD Systems, DY999) for 20–40 min at room temperature, protected from light. The color reaction was stopped by the addition of 50 μl 1 M H_2_SO_4_, and absorbance was read immediately using a plate reader at 450 nm (Labsystem Multiskan). The standard curve was calculated by plotting the standards against the absorbance values, and the cytokine levels were measured in picograms per millilitre.

### Western blot analysis

Mice whole brains were quickly dissected and frozen in liquid nitrogen and then kept at − 80 °C until used. The brains were homogenized in radioimmunoprecipitation assay (RIPA) lysis buffer (Thermo, 89900) using Qiagen Tissue lyser (85220) with metal beads for 30 s 3 to 4 cycles. The protein content was then analyzed with a bicinchoninic acid assay (BCA, Thermo Scientific, 23225). The samples were then equalized to 30 μg/well of protein with H_2_O. The samples were mixed with the sample buffer (Bio-Rad, 161-0737 supplemented with 10% β-mercaptoethanol, Sigma, M6250) and incubated for 10 min at 75 °C. The protein samples were then separated in 10% SDS-polyacrylamide gels and transferred onto a polyvinylidene membrane (Immobilon P, Millipore, IPVH00010). The membrane was blocked for 1 h at room temperature, incubated with primary antibody overnight at 4 °C, washed four times for 5 min, and then incubated with secondary antibody for 1 h at room temperature. Blocking and antibody incubation steps were performed in PBS supplemented with 0.1% Tween-20 (PBS-T) and 5% non-fat milk and wash steps using PBS-T. After further four 5-min washes, the membrane was incubated with chemiluminescent HRP substrate (Immobilon Western, Millipore, MDR-100-030Q) and read using Image Reader LAS-3000 (Fujifilm). Densitometry measurement of band intensity was used for quantification using ImageJ software. Bar graphs show mean relative density of the target protein to the reference protein.

### Immunocytochemistry

Immunocytochemistry of organotypic slices was performed by firstly washing the slices twice in PBS. The slices were then fixed and permeabilised by incubation in 4% paraformaldehyde (PFA) for 10 min on ice followed by incubation in ice-cold 100% methanol for 5 min. The slices were then washed twice for 5 min in PBS and incubated overnight in the TxBN buffer (PBS supplemented with 0.5% Triton-X100, 10% BSA, 1% normal goat serum (NGS, Abcam, ab7481)) to reduce nonspecific binding. For all antibody incubation and wash steps, TxB* buffer (PBS supplemented with 0.1% Triton-X and 2% BSA) was used. The slices were incubated overnight at room temperature with the primary antibodies, washed twice, and incubated overnight with secondary antibodies. The slices were then washed twice and mounted on glass cover slides with Tissue-Tek OCT Compound (Sakura, 4583). The slices were visualized with a Zeiss LSM 700 confocal microscope at various magnifications indicated in figure legends. Image stacks were initially used to analyze all layers of the slice and showed that optimal staining was confined to the central layers; images were thus taken from central layers within the tissue and then mean fluorescence intensity of MBP staining in the central white matter tracts was analyzed using ImageJ software. On average, four to six slices per condition were grown, six to eight images per slice were taken, and four regions per image were measured. Values in graphs show mean MBP fluorescence intensity of about 140 measurements with standard deviation of the mean (SD). For EBI2 internalization studies, U937 cells were washed once with PBS and incubated with 7α25HC (10 μM) or NIBR189 (10 μM) for 1 h at 37 °C. The U937 cells where then attached to cover slides using Thermo Scientific Cytospin 4 Cytocentrifuge (A78300003). All cells were washed with PBS and fixed with fixation/permeabilisation kit (BD Cytofix/Cytoperm, 554714) for 20 min on ice. Nonspecific binding was reduced by incubating cells for 1 h at room temperature in TwB buffer (PBS supplemented with 0.1% Tween-20, 1% BSA). For all antibody incubation and wash steps, TwB* (PBS supplemented with 0.05% Tween-20 and 0.5% BSA) was used. The cells were incubated overnight at 4 °C with the primary antibodies, washed twice, and incubated with secondary antibodies for 1 h. The cells were then washed twice, incubated with Hoechst in PBS for 10 min and washed twice again, and stored in PBS at 4 °C in the dark until imaged. The cells were visualized with a Zeiss LSM 700 confocal microscope.

## Results

### Organotypic cerebellar slices maintain host tissue cytoarchitecture and physiology

**Fig. 1:**
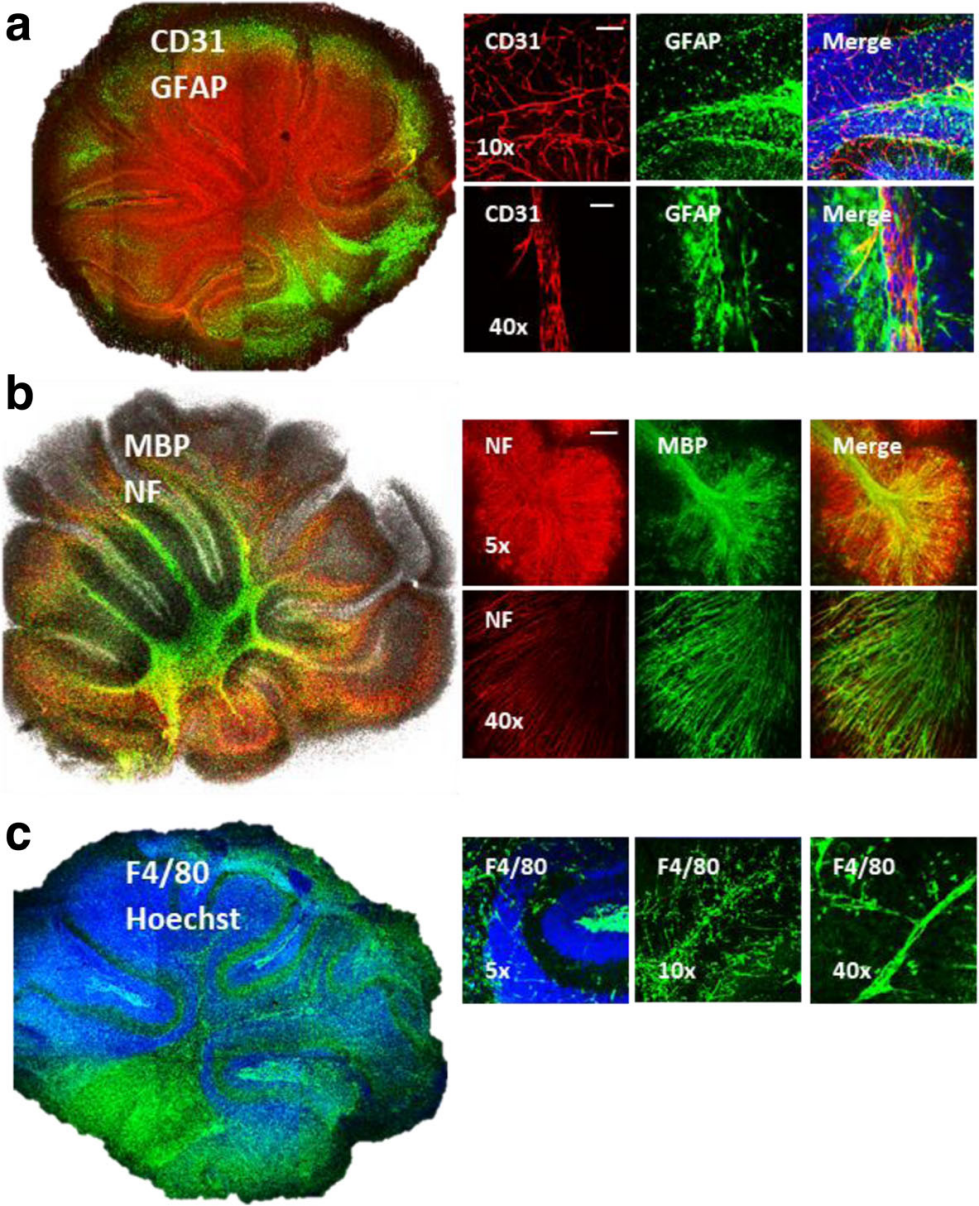
Organotypic cerebellar slices maintain host tissue cytoarchitecture and physiology. Confocal images show preserved cytoarchitecture in organotypic slice cultures prepared from P10 mice and cultured for 14 DIV. **a** Preserved blood vessel structure shown with CD31/PECAM-1 (red), endothelial cell marker. Images also show astrocyte (GFAP, green) staining throughout the entire slice as well as complete ensheathment of blood vessels with astrocytic end feet (higher magnification images). Nuclei stained with Hoechst (blue, merge image). **b** Images show a complete axon (NF, red) myelination with myelin (MBP, green). **c** Presence of immune cells shown here with anti-F4/80 antibody (green), a marker of microglia, mature macrophages, dendritic cells, and monocytes. Macrophages and monocytes align along the blood vessels as can be seen in higher magnification (×10 and ×40) images forming the BBB together with endothelial cells and astrocytes. Nuclei stained with Hoechst (blue, merge image). Images taken at ×10 (scale bar 100 μM), ×40 (scale bar 50 μM), and ×5 (scale bar 200 μM) magnification

The cytoarchitecture of cerebellar tissue in organotypic slices prepared from postnatal day 10 (P10) mice and cultured for 14 days in vitro (14 DIV) was investigated with immunocytochemistry. The data showed that CNS resident cells including neurons (Fig. 1 b), astrocytes (Fig. 1 a), microglia/macrophages (Fig. 1 c), and oligodendrocytes (Fig. 1 b) were present in the slices after 14 DIV. Peripheral immune cells expressing the F4/80 antigen such as macrophages, monocytes, and dendritic cells (Fig. 1 c) were also present in the slices. This data suggests, perhaps, that CNS-immune system interactions that occur in vivo may have potential to take place in these slice cultures. In addition, we find that microvascular morphology is also preserved as shown by alignment of CD31 (endothelial cells, Fig. 1 a), with GFAP (astrocytes, Fig. 1 a) and F4/80 (macrophages, Fig. 1 c), suggestive that GFAP and F4/80 positive cells envelop the CD31 cells in the brain as found in the blood-brain barrier (BBB). Most importantly, the data showed that myelination, which is mainly observed in the central white matter at P10, continues to develop in this in vitro model for at least 14 DIV [27–29, 35, 37] making it a model for studying myelination processes under normal and pathophysiological conditions (Fig. 1 b).

### EBI2 deficiency results in transient delay in MBP expression

**Fig. 2:**
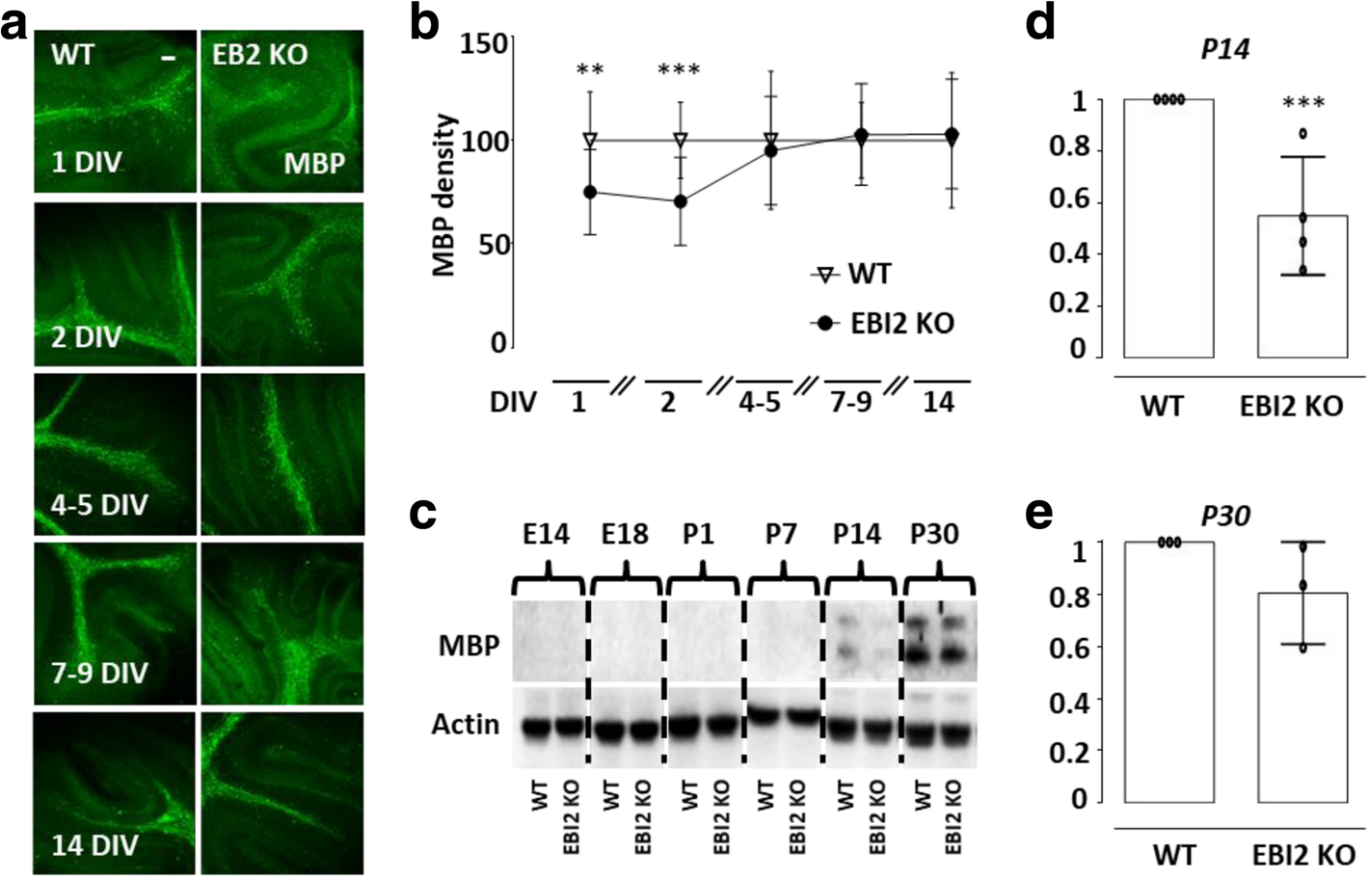
EBI2 deficiency results in transient delay in MBP expression. **a** Representative confocal images show mice cerebellar slices prepared from P10 mice and cultured for 1, 2, 4 to 5, 7 to 9, and 14 DIV. Myelin (MBP, green) staining is shown. Myelin development was determined by quantification of MBP staining density. Analysis showed significant differences in myelin development between one to two DIV. Images taken at ×10 magnification. Scale bar, 100 μM. **b** Graph showing quantification of MBP immunofluorescence in images such as those shown in (**a**). Data presented as mean ± SD (five to six animals per group), unpaired *t* test, **p < 0.01, ***p < 0.001 versus corresponding control. **c** Representative WBs show differential expression of MBP in whole brain lysates prepared from embryonic (E14 and E18) and postnatal (P1, P7, P14, and P30) WT and EBI2 KO mice. **d, e** Densitometric quantification of blots such as that shown in (**c**). Normalized WB data presented as mean ± SD (three to four WB data per time point; one animal per blot), unpaired *t* test, *** *p* < 0.001 versus corresponding control

Since oligodendrocytes use cholesterol as a building block for myelin and oxysterols are involved in cholesterol homeostasis and turnover, it is possible that activation of EBI2 by oxysterols plays an important role in myelination. In agreement with this idea, studies have shown that activation of LXR by oxysterols indeed regulates cholesterol homeostasis in oligodendrocytes [3]. Thus, to investigate whether EBI2 also has a role in regulation of myelination state, cerebellar slices were prepared from P10 EBI2 KO and WT mice and cultured for 1–14 DIV (Fig. 2 a). The extent of myelination was measured with confocal microscopy quantifying MBP staining. The data showed less MBP staining in cerebellar slices prepared from EBI2 KO mice compared to WT mice in the first one to two DIV (75.1 ± 20.6% at one DIV and 70.6 ± 21.3% at two DIV) and reaching WT levels after four to five DIV (95.1 ± 26.2%) (**p < 0.01, ***p < 0.001, five to six animals per group, independent Student *t* test) (Fig. 2 b). To further study the effects of EBI2 deficiency on the observed lower levels of MBP expression, whole mouse brain lysates of WT and EBI2 KO mice were prepared and MBP protein expression was measured with WB (Fig. 2 c–e). The analysis showed that MBP expression in brains from EBI2 KO mice is deficient at P14 compared to MBP expression in the brains from WT mice (54.9 ± 22.9%, ***p < 0.001, three animals per time point, independent Student *t* test) (Fig. 2 c, d). In agreement with organotypic slice data, MBP expression in vivo was deficient only at earlier stages of development and reached WT levels at P30 (80.5 ± 19.5%, *p* > 0.05, three animals per time point, independent Student *t* test) (Fig. 2 c, e). Interestingly, we have not observed differences in other myelin markers such as Olig1, CNPase, or MOG protein expression between WT and EBI2 KO mouse brains (Additional file 1: Fig. S1). The data suggest that EBI2 signaling is involved in myelin development at least during earlier stages of myelination and warrants further investigations into the role of EBI2 and oxysterols in myelination.

### Downregulation or antagonism of EBI2 signaling staggers myelination

**Fig. 3:**
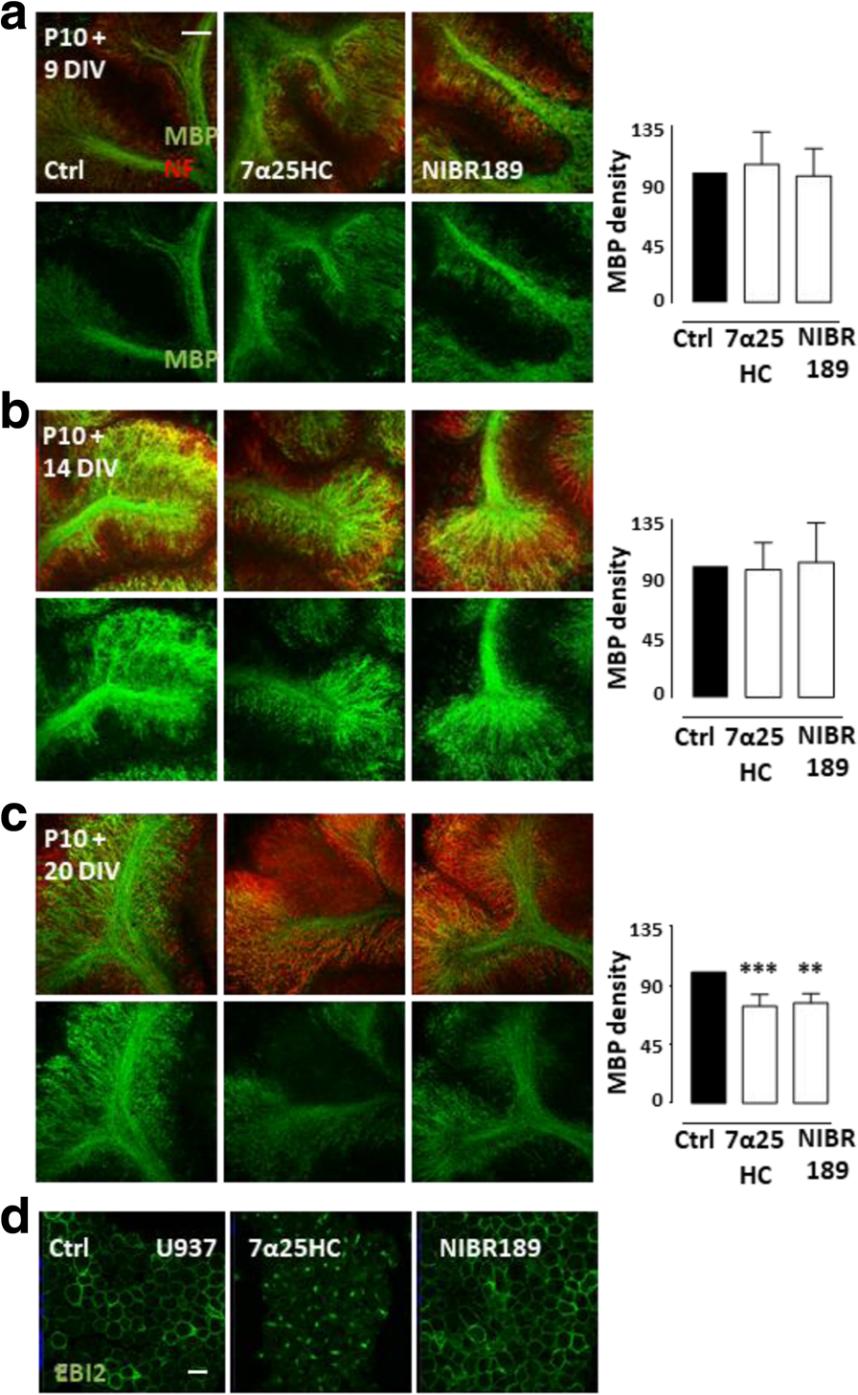
Downregulation or antagonism of EBI2 signaling staggers myelination. Representative confocal images show MBP expression (green) and NF light chain (red) in WT cerebellar slices prepared from P10 mice and cultured for **a** 9 DIV, **b** 14 DIV, and **c** 20 DIV with or without 7α25HC (1 μM) and NIBR189 (1 μM). Corresponding bar graphs shows quantification of MBP staining. There are no differences between non-treated and treated slices at 9 and 14 DIV. However, treatment of slices with 7α25HC or NIBR189 for 20 DIV inhibits normal myelin development in WT cerebellar slices as measured by MBP staining. Data presented as mean ± SD (*n* = 4 and 6), one-way ANOVA and Dunnett’s post-test, ** *p* > 0.01, *** *p* < 0.001 versus corresponding control. Images taken at ×10 magnification. Scale bar 100 μm (**d**). Treatment of human monocytes (U937) with 7α25HC (10 μM) for 1 h induces EBI2 (green) internalization. NIBR189 (10 μM) does not induce receptor internalization. Images were taken at ×40 magnification. Scale bar 20 μM

Myelin sheets are made up to a great extent from cholesterol and cholesterol derivatives; oxysterols are involved in cholesterol homeostasis [3, 38]. Cholesterol deficiency in myelinating oligodendrocytes can result in disrupted and delayed myelination showing that cholesterol is a crucial component of myelin. This lack of cholesterol can slow the rate of myelin formation and results in motor deficiency in mutant mice where myelinating oligodendrocytes cannot synthesize cholesterol [8]. To investigate the effects of long-term EBI2 agonism and antagonism on myelination, organotypic cerebellar slices were prepared from 10-day-old mice and cultured with or without the EBI2 agonist (7α25HC, 1 μM) or antagonist (NIBR189, 1 μM) for 9, 14, or 20 days in vitro (DIV) (Fig. 3). Neither agonism nor antagonism of EBI2 for 9 DIV (7α25HC 106.4 ± 24.5%, NIBR189 97.38 ± 21.1%, *p* > 0.05, *n* = 6, one-way ANOVA and Dunnett’s post-test) nor 14 DIV (7α25HC 97.3 ± 20.5%, NIBR189 103.0 ± 30.0%, p > 0.05, *n* = 4, one-way ANOVA and Dunnett’s post-test) induced changes in myelination compared to non-treated control as measured by MBP staining and immunocytochemistry (Fig. 3 a, b). However, long-term 20 DIV incubation of slices with the compounds induced a significant delay in MBP expression compared to non-treated controls (7α25HC 74.5 ± 8.8%, NIBR189 77.0 ± 7.1%, **p < 0.01, ***p < 0.001, *n* = 4, one-way ANOVA and Dunnett’s post-test) (Fig. 3 c). Interestingly, both agonism and antagonism of EBI2 induced similar inhibitory effect on MBP expression. To explain these apparently contradictory findings, we suggest that the EBI2 agonist 7α25HC is likely inducing receptor internalization and thus functional antagonistic effect as we have described for sphingosine 1-phosphate receptors (S1PR) [39, 40]. In agreement, we and others have observed that 7α25HC induces EBI2 receptor internalization in CHO-hEBI2 stable cells and in the U937 monocyte/macrophage cell line (Fig. 3 d) [33, 41, 42]. Taken together, the data showed that EBI2/oxysterol signaling may regulate myelination state.

### 7α25HC attenuates LPC-induced demyelination in mouse cerebellar slices

**Fig. 4:**
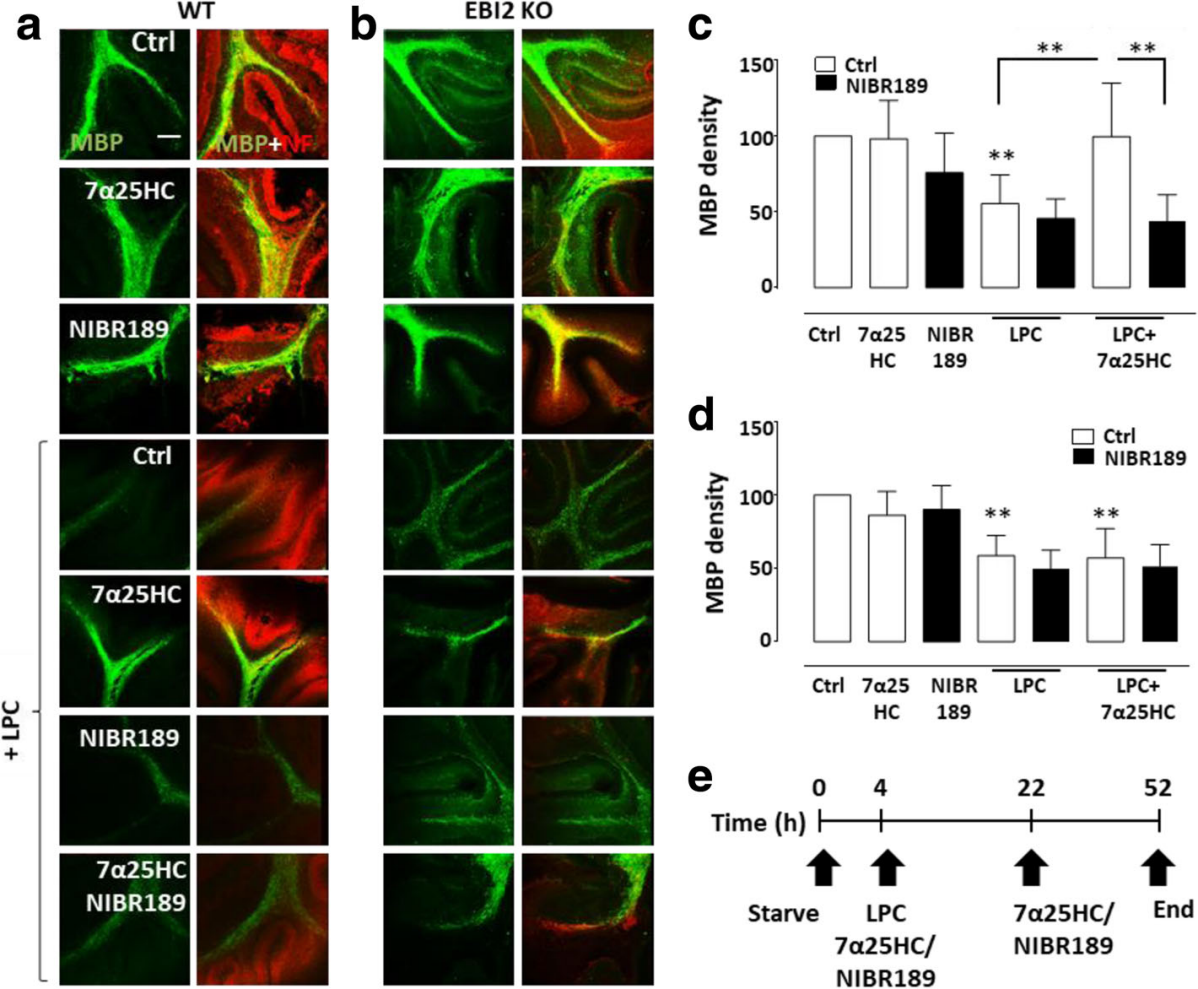
7α25HC protects from LPC-induced demyelination. Representative confocal images show WT (**a**) and EBI2 KO (**b**) mice cerebellar slices treated with 7α25HC (1 μM) and/or NIBR189 (1 μM) ± LPC (0.4 mg/ml). Anti-MBP (green) and anti-neurofilament (NF, red) immunostaining is shown. Images were taken at ×10 magnification. Scale bar, 100 μM. **c** A corresponding bar graph shows quantification of myelin staining in WT slices such as those shown in (**a**). 7α25HC significantly protects from LPC-induced demyelination, and this protective effect is inhibited with the EBI2 antagonist NIBR189 indicating EBI2 mediated protection from demyelination. Data presented as mean ± SD (*n* = 4 and 8), one-way ANOVA and Tukey’s multiple comparison post-test, ** *p* < 0.01 versus corresponding control. **d** A bar graph shows that 7α25HC does not protect from LPC-induced demyelination in slices prepared from EBI2 KO animals confirming EBI2-dependent mechanism shown in (**a** and **c**). Data presented as mean ± SD (*n* = 4), one-way ANOVA and Tukey’s multiple comparison post-test, **p < 0.01 versus control. **e** Schematic diagram illustrating experimental design

Studies have suggested that EBI2 and oxysterols are implicated in the pathogenesis of various autoimmune diseases [43, 44]. It has also been found that CH25H is up-regulated during demyelination as well remyelination in animal models of multiple sclerosis, EAE, to promote myelination [21]. In the current study, the effect of 7α25HC on LPC-induced demyelination was examined, similar to that we have described previously for the S1PR agonist, pFTY720 (Fig. 4) [27]. The effects of LPC and 7α25HC on myelination in slices prepared from WT mice (Fig. 4 a, c) and EBI2 KO mice (Fig. 4b, d) were investigated. In contrast to previous experiments, the slices were cultured for 14 DIV in complete media without the EBI2 compounds allowing them to myelinate fully before short-term treatments with LPC (18 h) and the compounds (48 h). Short-term treatment of fully myelinated WT slices with 7α25HC or NIBR189 alone had no significant effects on myelin state compared to control, as determined by MBP immunostaining (Fig. 4a, b). Importantly, 7α25HC (99.6 ± 35.1%) significantly protected from LPC-induced (55.4 ± 19.1%) demyelination in WT slices (**p < 0.01, *n* = 8, one-way ANOVA and Tukey’s post-test) (Fig. 4 c). Furthermore, the pre-treatment of slice cultures with EBI2 antagonist, NIBR189, reversed the protective effects of 7α25HC (44.1 ± 17.2%), indicating a receptor-dependent mechanism (**p < 0.01, *n* = 4, one-way ANOVA and Tukey’s post-test) (Fig. 4 c). To further confirm that the observed effects are EBI2 dependent, slices from EBI2 KO mice were treated same as above and immunostained for MBP (Fig. 4 b). The data showed that treatment with 7α25HC does not protect from LPC-induced demyelination (49.8 ± 12.7%) in EBI2 KO mice confirming EBI2-mediated effects (**p < 0.01, *n* = 4, one-way ANOVA, and Tukey’s post-test) (Fig. 4 d). Taken together, the data suggests that oxysterols as well as EBI2 signaling are involved in preserving myelination state under conditions of demyelination.

### EBI2 attenuates LPC-induced IL6 and IL1β release in mouse cerebellar slices

**Fig. 5:**
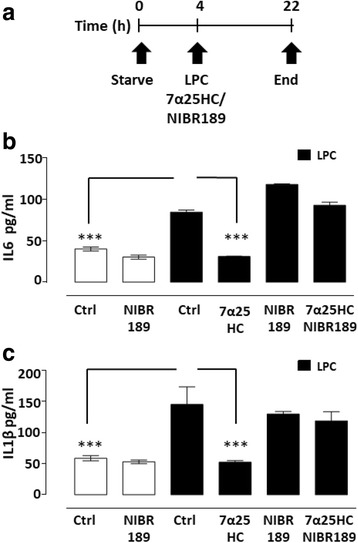
7α25HC attenuates LPC-induced levels of IL1β and IL6 in cerebellar slices. **a** Schematic diagram illustrating experimental design. Treatment of slices with LPC (0.4 mg/ml) induces an increase in IL6 (**b**) and IL1β (**c**) protein release. 7α25HC (1 μM) attenuates LPC (0.4 mg/ml)-induced levels of IL6 (**b**) and IL1β (**c**). Co-treatment with EBI2 antagonist (NIBR189, 1 μM) blocks the 7α25HC-mediated effects on the levels of IL6 (**b**) and IL1β (**c**) indicating EBI2-dependent regulation of cytokine release. Treatment with NIBR189 alone did not alter IL6 (**b**) or IL1β (**c**) release. Data presented as mean ± SD (*n* = 3), one-way ANOVA and Bonferroni’s post-test, *** *p* < 0.001 versus corresponding control

Studies have shown that oxysterols may exert anti-inflammatory effects inhibiting pro-inflammatory cytokine release [45–47]. For instance, it has been shown that mice deficient in the CH25H enzyme, which cannot synthesize oxysterol 25HC, show intensified EAE disease progress [18]. CH25H-deficient mouse macrophages also released greater amounts of IL1β after LPS stimulation than their wild type counterparts, indicating that 25HC has anti-inflammatory properties [18]. It has also been shown that pro-inflammatory cytokines can induce demyelination [24, 25]. To investigate whether LPC-induced demyelination was accompanied by increase in pro-inflammatory cytokines and whether EBI2 activation had an effect on cytokine release in these slices, media collected after 18 h treatment with LPC (0.4 μg/ml) with or without EBI2 agonist (7α25HC, 1 μM) or antagonist (NIBR189, 1 μM) was analyzed with IL6 and IL1β ELISAs (Fig. 5 a). Treatment with LPC alone significantly induced levels of IL6 (211.1 ± 6.1%) (Fig. 5 b) and IL1β (247.1 ± 48.1%) (Fig. 5 c) in cerebellar slices after 18 h (***p < 0.001, *n* = 3, one-way ANOVA and Bonferroni post-test). Treatment with NIBR189 (75.6 ± 5.9% for IL6, 90.0 ± 5.2% for IL1β) alone had no effect on IL6 and IL1β release from organotypic slices (Fig. 5 b, c) (*p* > 0.05, *n* = 3, one-way ANOVA and Bonferroni post-test). However, pre-treatment of slices with 7α25HC attenuated the LPC-induced levels of IL6 (36.5 ± 0.7%) (Fig. 5b) and IL1β (36.0 ± 1.8%) (Fig. 5c) (***p < 0.001, *n* = 3, one-way ANOVA and Bonferroni post-test). Moreover, demonstrating EBI2-dependent mechanism, NIBR189 blocked the anti-inflammatory effects of 7α25HC on LPC-induced levels of IL6 (109.7 ± 4.5%) (Fig. 5b) and IL1β (81.8 ± 10.2%) (Fig. 5c) (*p* > 0.05 versus LPC, *n* = 3, one-way ANOVA and Bonferroni post-test). In summary, the data shows that EBI2 and oxysterols are involved in regulation of normal myelination as well as protection during inflammatory processes possibly via inhibition of pro-inflammatory cytokine levels.

## Discussion

EBI2 is a G protein-coupled receptor that plays an important role in orchestrating T cell-dependent antibody response in lymphoid tissue [48, 49]. The oxysterol 7α25HC is the most potent known endogenous agonist for EBI2 [33, 41]. Oxysterols are derivatives of cholesterol which, among other functions, also act as immune regulators [38]. A large quantity of cholesterol is located in the brain where it serves as a building block for myelin sheets of oligodendrocytes [50]. Oxysterol signaling has been implicated in certain diseases of the CNS with immune and demyelinating component, although with somewhat opposite effects depending on the study [14–16, 19, 20, 51, 52]. For instance, anti-inflammatory and disease-alleviating properties of oxysterol signaling have been reported in microglia during demyelinating and remyelinating phases of EAE [21]. In another study, CH25H-deficient mice showed exaggerated EAE progression and intensified pro-inflammatory cytokine profile [18]. Reduced levels of 25CH have also been found in relapsing-remitting MS patients [14]. Mutation in another enzyme needed for 7α25HC synthesis, namely CYP7B1, is the cause of a neurodegenerative disorder called spastic paraplegia type 5 (SPG5), which is characterized by white matter lesions [22, 23]. However, contrary findings were reported showing attenuated disease course in EAE in CH25H KO animals [19] and in mice injected with 25HC as well as cells derived from childhood cerebral X-linked adrenoleukodystrophy patients [20]. Moreover, in EAE, expression of EBI2 receptor in Th17 cells, CH25H in microglia and CYP7B1 in infiltrating immune cells have been found to be increased demonstrating EBI2/oxysterol role as a mediator of autoimmunity and inflammation [16]. Increased expression of EBI2 and oxysterols has been found in MS lesions and specifically in infiltrating cells such as CD4+ T cells possibly indicating disease aggravating signaling [15, 16]. In light of the above often contradictory findings, further elucidation of EBI2/oxysterols signaling in CNS immune defense and its potential involvement in myelination is needed. Here, the effects of EBI2 on normal myelin development and demyelination under pathophysiological conditions were investigated. It was demonstrated that myelin in EBI2 KO mice develops slower than in WT mice. In agreement with the knock-out studies, pharmacological studies showed that long-term inhibition of EBI2 signaling either via persistent antagonism or receptor internalization and downregulation staggers myelin development. In addition to regulation of myelin development under normal conditions, the data showed that activation of EBI2 protects from LPC-induced demyelination and that the protective effects might be mediated via inhibition of pro-inflammatory cytokine release.

### EBI2 is involved in myelin development

Studies have shown the importance of high cholesterol synthesis and homeostasis for proper myelin development [8]. Abnormalities in cholesterol or oxysterol levels have also been linked to defective myelination [8–10]. In the current study, investigations of the expression of myelin proteins in WT and EBI2 KO mice at various stages of development showed that MBP expression in the brains of EBI2 KO mice is delayed compared to WT mice. Notably, a similar pattern of MBP expression was observed in vivo and in vitro. MBP expression was reduced in cerebellar slices prepared from P10 EBI2 KO mice grown in vitro for 1–14 days compared to slices prepared from WT mice. The data showed an initial delay of MBP expression and thereafter an eventual MBP recovery in EBI2 KO as well as in organotypic slices treated with EBI2 compounds. MBP is thought to be involved in myelin compaction during oligodendrocyte maturation [53]. In shiverer mice, where the MBP gene has been mutated, formation of the major dense line in compact myelin is affected [54, 55]. It is therefore possible that EBI2 signaling is involved in later stages of myelin development when myelin is being compacted. At this stage of myelination, an asymmetry in lipid composition during myelin compaction is being formed [56]. The glycolipids are positioned in the outer surface in the intraperiodic line, and negatively charged phospholipids are located on the inner part [56]. Given that we find EBI2 regulates MBP levels, this receptor might play an important role in the process of compaction. Interestingly, we have not observed differences in Olig1, CNPase, or MOG protein expression between WT and EBI2 KO mouse brains. These and other markers are expressed at various stages of oligodendrocyte development to guide the maturation of myelin. The lack of differences in the expression of these proteins, except MBP, in WT and EBI2 KO animals indicates that EBI2 signaling is not involved at all stages of myelin development. This may also suggest that EBI2 may only be involved in regulation of fine-tuning of myelin content and compaction.

### Inhibition of EBI2 signaling leads to delay in MBP expression

The data from pharmacological in vitro studies confirmed the findings from the in vivo and in vitro genetic knock-out experiments. Both, long-term EBI2 antagonism and agonism induced a delay in MBP expression. The EBI2 agonist, 7α25HC, has been shown to induce receptor internalization in CHO cells stably expressing human EBI2 and in the U937 monocyte/macrophage cell line [33, 41, 42]. It is therefore possible that long-term EBI2 agonism led to receptor internalization and its subsequent downregulation on the cell membrane inducing a functional antagonist effect as has previously been described for S1P1 receptors [39, 40]. The effects of the EBI2 compounds on MBP expression was observed after as long as 20 DIV and was not present at earlier time points. The differences in myelination might not have been observed at earlier stages due to the fact that EBI2 signaling was present up to P10 at which stage the slices were prepared and maintained in media supplemented with the EBI2 compounds. Thus, it is conceivable that the MBP expression continued for some time after the signal was interrupted pharmacologically with EBI2 compounds resulting in the slowing down of MBP expression.

### EBI2 protects from LPC-induced demyelination via inhibition of pro-inflammatory cytokine release

Lastly, the current study demonstrated that activation of EBI2 can attenuate LPC-induced demyelination in WT mouse cerebellar organotypic slice cultures, but not in those prepared from EBI2 KO mice. It also showed that a reduction in the levels of pro-inflammatory cytokines may play a role in these protective effects. Moreover, the EBI2 antagonist inhibited the effects of 7α25HC on LPC-induced demyelination in organotypic mice cerebellar slice cultures. These protective effects of 7α25HC were lost in slices prepared from EBI2 KO animals. Of interest, the effects of 7α25HC on attenuating LPC-induced demyelination in organotypic mice cerebellar slice cultures resembled the effects of the S1P1 receptor modulator pFTY720 in our previous studies [27]. These findings are in agreement with recent findings in the EAE model where CH25H (enzyme needed for 25HC synthesis)-deficient mice exhibited exaggerated course of the disease probably due to the diminished negative feedback loop exerted by 25HC on the IL1-family of cytokines [18]. However, it needs to be noted that our observations are not in agreement with other studies which found that CH25H KO mice show an alleviated course of EAE [19] and that WT mice expression of CH25H, CYP7B1, and the receptor itself is increased leading to autoimmunity and migration of reactive T cells into inflamed tissue [16]. Overall, these experiments provide genetic and pharmacological support for an EBI2-mediated process during CNS inflammation and demyelination. More research is needed, however, to elucidate the exact role and significance of the receptor and its ligand in these disease processes.

## Conclusions

Given the data presented in the current study, we are now examining the effects of EBI2 deficiency in in vivo models of MS, namely in the LPS-challenge and cuprizone models. The value of using the EBI2-null animals in these models of MS is important to determine the value of this receptor as a drug target in demyelinating illnesses such as MS. It is equally interesting to note that mutations in CYP7B1 have been identified in patients with an autosomal recessive form of hereditary spastic paraplegia called SPG5 (for a recent review see [57]). Several groups reported white-matter MRI abnormalities indicative of possible demyelination in cases of SPG5 [23, 58, 59]. These results suggest further that reduced levels of EBI2-activating oxysterols may alter myelination state. Moreover, these genetic findings support the idea that activation of EBI2 could be important for therapeutic intervention in selected patients suffering from SPG5. In closing, we suggest this work and further in vivo studies will support the use of EBI2 as a novel drug target for treatment of MS and possibly other neuroinflammatory and neurodegenerative diseases.

## Additional file

Additional file 1: Figure S1. Olig1, CNPase, and MOG are not differentially expressed in WT and EBI2 KO mice. (a) Representative WBs show quantitative differences in oligodendrocyte marker expression in whole brain lysates prepared from embryonic (E14 and E18) and postnatal (P1, P7, P14,and P30) mouse brains. Densitometric quantification of blots shown in (a) indicating that Olig1 (b) CNPase (c) and MOG (d) expression is not different in WT and EBI2 KO animals. Data presented as single measurement, three animals per time point. (PDF 468 kb)

## Abbreviations

7α25HC: 7α, 25-dihydroxycholesterol
EAE: Experimental autoimmune encephalomyelitis
EBI2: Epstein-Barr virus-induced gene 2
KO: Knock-out
LPC: Lysolecithin
MS: Multiple sclerosis
WT: Wild type

## References

[1] Gatto D, Wood K, Brink R (2011). J Immunol.

[2] Pereira JP, Kelly LM, Cyster JG (2010). Finding the right niche: B-cell migration in the early phases of T-dependent antibody responses. Int Immunol.

[3] Nelissen K, Mulder M, Smets I, Timmermans S, Smeets K, Ameloot M, Hendriks JJ, Liver X (2011). Receptors regulate cholesterol homeostasis in oligodendrocytes. J Neurosci Res.

[4] Cyster JG, Dang EV, Reboldi A, Yi T (2014). 25-Hydroxycholesterols in innate and adaptive immunity. Nat Rev Immunol.

[5] Rutkowska A, Preuss I, Gessier F, Sailer AW, Dev KK (2015). EBI2 regulates intracellular signaling and migration in human astrocyte. Glia.

[6] Rutkowska A, O'Sullivan SA, Christen I, Zhang J, Sailer AW, Dev KK (2016). The EBI2 signalling pathway plays a role in cellular crosstalk between astrocytes and macrophages. Sci Rep.

[7] Rutkowska A, Dev KK, Sailer AW (2016). The role of the oxysterol/EBI2 pathway in the immune and central nervous systems. Curr Drug Targets.

[8] Saher G, Brugger B, Lappe-Siefke C, Mobius W, Tozawa R, Wehr MC, Wieland F, Ishibashi S, Nave KA (2005). High cholesterol level is essential for myelin membrane growth. Nat Neurosci.

[9] Makoukji J, Shackleford G, Meffre D, Grenier J, Liere P, Lobaccaro JM, Schumacher M, Massaad C (2011). Interplay between LXR and Wnt/beta-catenin signaling in the negative regulation of peripheral myelin genes by oxysterols. J Neurosci.

[10] Trousson A, Bernard S, Petit PX, Liere P, Pianos A, El Hadri K, Lobaccaro JM, Ghandour MS, Raymondjean M, Schumacher M, Massaad C (2009). 25-hydroxycholesterol provokes oligodendrocyte cell line apoptosis and stimulates the secreted phospholipase A2 type IIA via LXR beta and PXR. J Neurochem.

[11] Lutjohann D, Papassotiropoulos A, Bjorkhem I, Locatelli S, Bagli M, Oehring RD, Schlegel U, Jessen F, Rao ML, von Bergmann K, Heun R (2000). Plasma 24S-hydroxycholesterol (cerebrosterol) is increased in Alzheimer and vascular demented patients. J Lipid Res.

[12] Leoni V, Masterman T, Diczfalusy U, De Luca G, Hillert J, Bjorkhem I (2002). Changes in human plasma levels of the brain specific oxysterol 24S-hydroxycholesterol during progression of multiple sclerosis. Neurosci Lett.

[13] Panzenboeck U, Andersson U, Hansson M, Sattler W, Meaney S, Bjorkhem I (2007). On the mechanism of cerebral accumulation of cholestanol in patients with cerebrotendinous xanthomatosis. J Lipid Res.

[14] Crick PJ, Griffiths WJ, Zhang J, Beibel M, Abdel-Khalik J, Kuhle J, Sailer AW, Wang Y (2016). Reduced plasma levels of 25-hydroxycholesterol and increased cerebrospinal fluid levels of bile acid precursors in multiple sclerosis patients. Mol Neurobiol.

[15] Clottu AS, Mathias A, Sailer AW, Schluep M, Seebach JD, Du Pasquier R (2017). Pot C. EBI2 expression and function: robust in memory lymphocytes and increased by natalizumab in multiple sclerosis. Cell Rep.

[16] Wanke F, Moos S, Croxford AL, Heinen AP, Graf S, Kalt B, Tischner D, Zhang J, Christen I, Bruttger J (2017). EBI2 is highly expressed in multiple sclerosis lesions and promotes early CNS migration of encephalitogenic CD4 T cells. Cell Rep.

[17] Diestel A, Aktas O, Hackel D, Hake I, Meier S, Raine CS, Nitsch R, Zipp F, Ullrich O (2003). Activation of microglial poly(ADP-ribose)-polymerase-1 by cholesterol breakdown products during neuroinflammation: a link between demyelination and neuronal damage. J Exp Med.

[18] Reboldi A, Dang EV, McDonald JG, Liang G, Russell DW, Cyster JG (2014). 25-Hydroxycholesterol suppresses interleukin-1–driven inflammation downstream of type I interferon. Science.

[19] Chalmin F, Rochemont V, Lippens C, Clottu A, Sailer AW, Merkler D, Hugues S, Pot C (2015). Oxysterols regulate encephalitogenic CD4(+) T cell trafficking during central nervous system autoimmunity. J Autoimmun.

[20] Jang J, Park S, Jin Hur H, Cho HJ, Hwang I, Pyo Kang Y, Im I, Lee H, Lee E, Yang W (2016). 25-hydroxycholesterol contributes to cerebral inflammation of X-linked adrenoleukodystrophy through activation of the NLRP3 inflammasome. Nat Commun.

[21] Olah M, Amor S, Brouwer N, Vinet J, Eggen B, Biber K, Boddeke HW (2011). Identification of a microglia phenotype supportive of remyelination. Glia.

[22] Schule R, Schols L (2011). Genetics of hereditary spastic paraplegias. Semin Neurol.

[23] Biancheri R, Ciccolella M, Rossi A, Tessa A, Cassandrini D, Minetti C, Santorelli FM (2009). White matter lesions in spastic paraplegia with mutations in SPG5/CYP7B1. Neuromuscul Disord.

[24] Redford EJ, Hall SM, Smith KJ (1995). Vascular changes and demyelination induced by the intraneural injection of tumour necrosis factor. Brain.

[25] Ferrari CC, Depino AM, Prada F, Muraro N, Campbell S, Podhajcer O, Perry VH, Anthony DC, Pitossi FJ (2004). Reversible demyelination, blood-brain barrier breakdown, and pronounced neutrophil recruitment induced by chronic IL-1 expression in the brain. Am J Pathol.

[26] di Penta A, Moreno B, Reix S, Fernandez-Diez B, Villanueva M, Errea O, Escala N, Vandenbroeck K, Comella JX, Villoslada P (2013). Oxidative stress and proinflammatory cytokines contribute to demyelination and axonal damage in a cerebellar culture model of neuroinflammation. PLoS One.

[27] Sheridan GK, Dev KK (2012). S1P1 receptor subtype inhibits demyelination and regulates chemokine release in cerebellar slice cultures. Glia.

[28] O'Sullivan C, Schubart A, Mir AK, Dev KK (2016). The dual S1PR1/S1PR5 drug BAF312 (Siponimod) attenuates demyelination in organotypic slice cultures. J Neuroinflammation.

[29] O'Sullivan SA, Velasco-Estevez M, Dev KK (2017). Demyelination induced by oxidative stress is regulated by sphingosine 1-phosphate receptors. Glia.

[30] Kim HJ, Miron VE, Dukala D, Proia RL, Ludwin SK, Traka M, Antel JP, Soliven B (2011). Neurobiological effects of sphingosine 1-phosphate receptor modulation in the cuprizone model. FASEB J.

[31] Gessier F, Preuss I, Yin H, Rosenkilde MM, Laurent S, Endres R, Chen YA, Marsilje TH, Seuwen K, Nguyen DG, Sailer AW (2014). Identification and characterization of small molecule modulators of the Epstein-Barr virus-induced gene 2 (EBI2) receptor. J Med Chem.

[32] Costagliola S, Many MC, Denef JF, Pohlenz J, Refetoff S, Vassart G (2000). Genetic immunization of outbred mice with thyrotropin receptor cDNA provides a model of Graves’ disease. J Clin Invest.

[33] Hannedouche S, Zhang J, Yi T, Shen W, Nguyen D, Pereira JP, Guerini D, Baumgarten BU, Roggo S, Wen B (2011). Oxysterols direct immune cell migration via EBI2. Nature.

[34] Pritchard AJ, Mir AK, Dev KK (2014). Fingolimod attenuates splenocyte-induced demyelination in cerebellar slice cultures. PLoS One.

[35] O'Sullivan C, Dev KK (2015). Galactosylsphingosine (psychosine)-induced demyelination is attenuated by sphingosine 1-phosphate signalling. J Cell Sci.

[36] Elain G, Jeanneau K, Rutkowska A, Mir AK, Dev KK (2014). The selective anti-IL17A monoclonal antibody secukinumab (AIN457) attenuates IL17A-induced levels of IL6 in human astrocytes. Glia.

[37] Birgbauer E, Rao TS, Webb M (2004). Lysolecithin induces demyelination in vitro in a cerebellar slice culture system. J Neurosci Res.

[38] Russell DW (2000). Oxysterol biosynthetic enzymes. Biochim Biophys Acta.

[39] Mullershausen F, Zecri F, Cetin C, Billich A, Guerini D, Seuwen K (2009). Persistent signaling induced by FTY720-phosphate is mediated by internalized S1P1 receptors. Nat Chem Biol.

[40] Healy LM, Sheridan GK, Pritchard AJ, Rutkowska A, Mullershausen F, Dev KK (2013). Pathway specific modulation of S1P1 receptor signalling in rat and human astrocytes. Br J Pharmacol.

[41] Liu C, Yang XV, Wu J, Kuei C, Mani NS, Zhang L, Yu J, Sutton SW, Qin N, Banie H (2011). Oxysterols direct B-cell migration through EBI2. Nature.

[42] Yi T, Wang X, Kelly LM, An J, Xu Y, Sailer AW, Gustafsson JA, Russell DW, Cyster JG (2012). Oxysterol gradient generation by lymphoid stromal cells guides activated B cell movement during humoral responses. Immunity.

[43] Ye S, Pang H, YY G, Hua J, Chen XG, Bao CD, Wang Y, Zhang W, Qian J, Tsao BP (2003). Protein interaction for an interferon-inducible systemic lupus associated gene, IFIT1. Rheumatology (Oxford).

[44] Heinig M, Petretto E, Wallace C, Bottolo L, Rotival M, Lu H, Li Y, Sarwar R, Langley SR, Bauerfeind A (2010). A trans-acting locus regulates an anti-viral expression network and type 1 diabetes risk. Nature.

[45] Englund MC, Karlsson AL, Wiklund O, Bondjers G, Ohlsson BG (2001). 25-hydroxycholesterol induces lipopolysaccharide-tolerance and decreases a lipopolysaccharide-induced TNF-alpha secretion in macrophages. Atherosclerosis.

[46] Ohlsson BG, Englund MC, Karlsson AL, Knutsen E, Erixon C, Skribeck H, Liu Y, Bondjers G, Wiklund O (1996). Oxidized low density lipoprotein inhibits lipopolysaccharide-induced binding of nuclear factor-kappaB to DNA and the subsequent expression of tumor necrosis factor-alpha and interleukin-1beta in macrophages. J Clin Invest.

[47] Moog C, Luu B, Beck JP, Italiano L, Bischoff P (1988). Studies on the immunosuppressive properties of 7,25-dihydroxycholesterol—I. Reduction of interleukin production by treated lymphocytes. Int J Immunopharmacol.

[48] Gatto D, Paus D, Basten A, Mackay CR, Brink R (2009). Guidance of B cells by the orphan G protein-coupled receptor EBI2 shapes humoral immune responses. Immunity.

[49] Kelly LM, Pereira JP, Yi T, Xu Y, Cyster JG (2011). EBI2 guides serial movements of activated B cells and ligand activity is detectable in lymphoid and nonlymphoid tissues. J Immunol.

[50] Bjorkhem I, Meaney S (2004). Brain cholesterol: long secret life behind a barrier. Arterioscler Thromb Vasc Biol.

[51] Leoni V, Caccia C (2011). Oxysterols as biomarkers in neurodegenerative diseases. Chem Phys Lipids.

[52] Leoni V (2009). Oxysterols as markers of neurological disease—a review. Scand J Clin Lab Invest.

[53] Campagnoni AT, Macklin WB (1988). Cellular and molecular aspects of myelin protein gene expression. Mol Neurobiol.

[54] Privat A, Jacque C, Bourre JM, Dupouey P, Baumann N (1979). Absence of the major dense line in myelin of the mutant mouse “shiverer”. Neurosci Lett.

[55] Roach A, Takahashi N, Pravtcheva D, Ruddle F, Hood L (1985). Chromosomal mapping of mouse myelin basic protein gene and structure and transcription of the partially deleted gene in shiverer mutant mice. Cell.

[56] Baumann N, Pham-Dinh D (2001). Biology of oligodendrocyte and myelin in the mammalian central nervous system. Physiol Rev.

[57] Schule R, Siddique T, Deng HX, Yang Y, Donkervoort S, Hansson M, Madrid RE, Siddique N, Schols L, Bjorkhem I (2010). Marked accumulation of 27-hydroxycholesterol in SPG5 patients with hereditary spastic paresis. J Lipid Res.

[58] Criscuolo C, Filla A, Coppola G, Rinaldi C, Carbone R, Pinto S, Wang Q, de Leva MF, Salvatore E, Banfi S (2009). Two novel CYP7B1 mutations in Italian families with SPG5: a clinical and genetic study. J Neurol.

[59] Goizet C, Boukhris A, Durr A, Beetz C, Truchetto J, Tesson C, Tsaousidou M, Forlani S, Guyant-Marechal L, Fontaine B (2009). CYP7B1 mutations in pure and complex forms of hereditary spastic paraplegia type 5. Brain.

