# Supplementary figures and images for: EBI2 receptor regulates myelin development and inhibits LPC-induced demyelination
Source: J Neuroinflammation. 2017 Dec 16;14:250. doi: 10.1186/s12974-017-1025-0 (PMC5732472; doi:10.1186/s12974-017-1025-0)

## Supplemental Figure 1

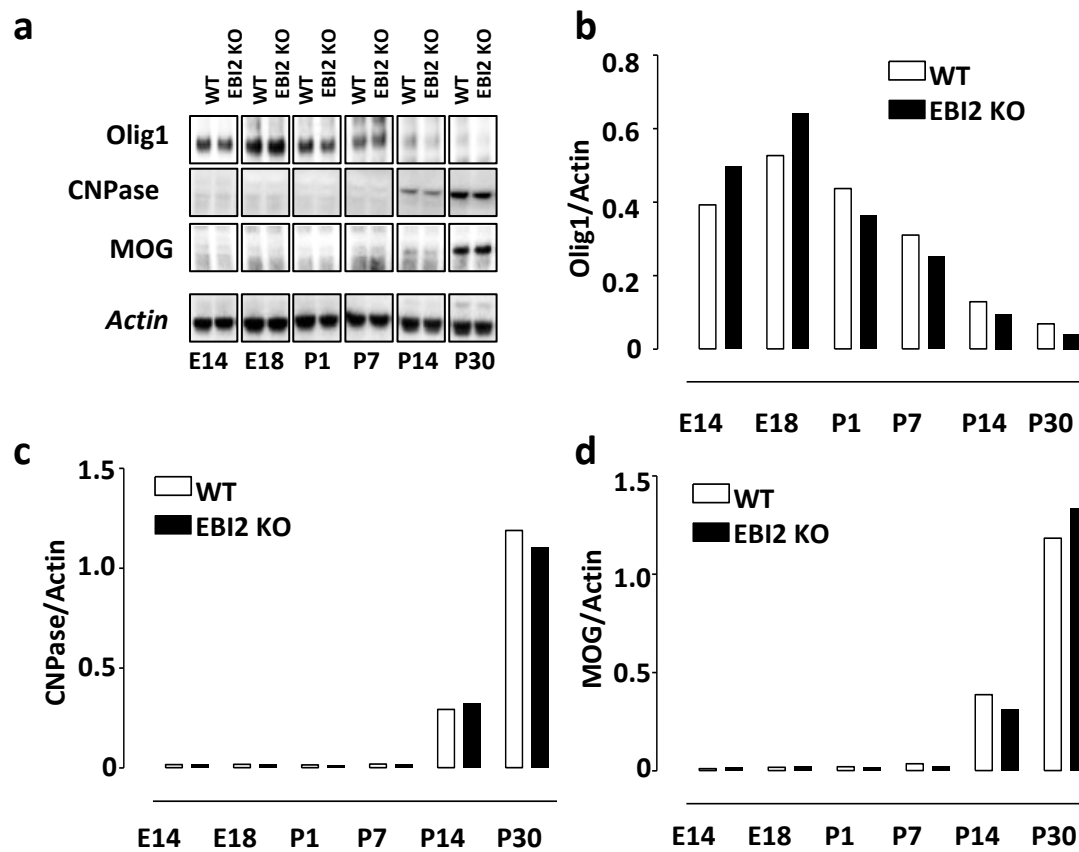

Supplement: Additional file 1: Figure S1. — Olig1, CNPase, and MOG are not differentially expressed in WT and EBI2 KO mice. (a) Representative WBs show quantitative differences in oligodendrocyte marker expression in whole brain lysates prepared from embryonic (E14 and E18) and postnatal (P1, P7, P14,and P30) mouse brains. Densitometric quantification of blots shown in (a) indicating that Olig1 (b) CNPase (c) and MOG (d) expression is not different in WT and EBI2 KO animals. Data presented as single measurement, three animals per time point. (PDF 468 kb) [file 12974_2017_1025_MOESM1_ESM.pdf]
